# New roles for glutathione: Modulators of bacterial virulence and pathogenesis

**DOI:** 10.1016/j.redox.2021.102012

**Published:** 2021-05-29

**Authors:** Joanne Wei Kay Ku, Yunn-Hwen Gan

**Affiliations:** a Infectious Diseases Translational Research Program, Yong Loo Lin School of Medicine, National University of Singapore, Singapore; b Department of Biochemistry, National University of Singapore, 8 Medical Drive, 117596, Singapore

**Keywords:** Glutathione, Virulence, Bacteria, Pathogenesis, Thiol, Immune response

## Abstract

Low molecular weight (LMW) thiols contain reducing sulfhydryl groups that are important for maintaining antioxidant defense in the cell. Aside from the traditional roles of LMW thiols as redox regulators in bacteria, glutathione (GSH) has been reported to affect virulence and bacterial pathogenesis. The role of GSH in virulence is diverse, including the activation of virulence gene expression and contributing to optimal biofilm formation. GSH can also be converted to hydrogen sulfide (H_2_S) which is important for the pathogenesis of certain bacteria. Besides GSH, some bacteria produce other LMW thiols such as mycothiol and bacillithiol that affect bacterial virulence. We discuss these newer reported functions of LMW thiols modulating bacterial pathogenesis either directly or indirectly and via modulation of the host immune system.

## 1 Introduction

Low molecular weight (LMW) thiols are molecules containing reducing sulfhydryl groups that enable the detoxification of reactive oxygen species (ROS), reactive nitrogen species (RNS) and other free radicals. LMW thiols are involved in a range of biological functions including antioxidant defense as well as cell signaling and the modulation of the immune system in eukaryotes [1,2]. In bacteria, LMW thiols can contribute to fitness and survival in adverse conditions such as countering oxidative stress as well as modulating pathogenesis [[3], [4], [5]]. Although the role of LMWs is traditionally viewed as redox regulators in bacteria, there is a slow but steady increase of reports indicating that LMW thiols can alter bacterial pathogenesis in more direct ways. The predominant LMW thiol in Gram-negative bacteria is glutathione (GSH; L-γ-glutamyl-L-cysteinyl-glycine) with concentrations in the millimolar range [6]. Only a few Gram-positive bacteria, such as *Listeria monocytogenes* and *Streptococcus agalacti*ae, produce GSH [7,8].

Redox function of LMW thiols affecting bacterial fitness has been comprehensively discussed elsewhere [2]. In this review, we discuss the influence of LMW thiols on bacterial virulence during infection. However, some virulence functions described here could still be indirectly due to a compromised redox balance affecting bacterial fitness, as many studies show how the lack of GSH affects a phenotype associated with virulence, without defining how GSH is doing so mechanistically. We focus on the major thiols present in bacteria including GSH, widely present in Gram-negative bacteria, mycothiol (MSH), the major thiol in Actinobacteria, and bacillithiol (BSH), found in many Gram-positive bacteria.

## 2 GSH as a virulence switch in bacteria

GSH has not been thought of as a signal used by bacteria for turning on or off virulence till in 2015 following two separate reports in *Burkholderia pseudomallei* and *Listeria monocytogenes* [9,10].

*B. pseudomallei* is a facultative intracellular bacterium with a broad host range and with the ability to infect many eukaryotic cells [[11], [12], [13]]. Upon invasion or uptake into host cells, *B. pseudomallei* escapes from the phagosome into the host cytosol mediated by the Type 3 Secretion System 3 (T3SS3), a bacterial secretion system found in many pathogenic Gram-negative bacteria that is able to secrete effectors into host cells to interfere with host cell functions [14,15]. *B. pseudomallei* T3SS3 is expressed once the bacteria contact host cells [16], while another secretion system, Type 6 Secretion System 5 (T6SS5), is only expressed when bacteria are intracellular [17]. T6SS5 is necessary for *B. pseudomallei* to fuse host cells together in the formation of multinucleated giant cells (MNGCs) and this facilitates bacterial spreading from cell-to-cell [18]. Both T3SS3 and T6SS5 are critical for the pathogenesis of *B. pseudomallei* in mice, where either T3SS3 or T6SS5 mutants are avirulent [17,19]. An intriguing question that arises is how *B. pseudomallei* can sense its intracellular environment in order to turn on T6SS5 gene transcription. When exogenous GSH, but not GSSG, was added to *B. pseudomallei* grown in medium, its T6SS5 gene expression was upregulated significantly, to more than 100-fold. Inside infected RAW264.7 macrophages, both wildtype *B. pseudomallei* and the mutant defective in GSH synthesis (Δ*gshB*) were able to upregulate T6SS5 gene expression to the same extent. Furthermore, HepG2 mutant cell line that was grown in cysteine-deficient medium or peripheral blood mononuclear cells (PBMCs) depleted of GSH via diethyl maleate (DEM) showed decreased T6SS5 gene expression during bacterial infection [10]. These findings confirmed that host GSH, and not bacterial GSH, is mediating T6SS5 gene expression. When the bacterium exits the oxidizing phagosome and into the cytosol, it is suddenly exposed to a reducing environment due to the high concentrations of host GSH [10]. GSH entry into the bacterial periplasm through a yet undefined mechanism, reduces VirA, the histidine kinase sensor protein present on the inner membrane of the bacteria. The reduction of VirA occurs on a periplasmically located cysteine residue, resulting in the switch from a dimeric to a monomeric form [10]. The monomeric VirA triggers T6SS5 expression through VirG, its DNA response regulator [17]. Thus, *B. pseudomallei* senses its entry into the cytosol through the action of GSH on VirA and VirG, the two-component sensor regulator for T6SS5 gene expression [10]. This fascinating system uncovers how *B. pseudomallei* is able to exploit host GSH as a spatio-temporal cue to transcriptionally turn on its T6SS5 cluster at the precise moment for intercellular spreading only when the bacterium has exited the phagosome [3].

Although Gram-positive, *L. monocytogenes* synthesizes GSH. GSH synthase (*gshF*) mutants have downregulated virulence gene expression and are less virulent in mice [9]. It shares certain virulence features as *B. pseudomallei* such as being facultative intracellular and the ability to polymerize actin on one bacterial pole to propel bacterial movement intercellularly. It has been previously reported to utilize GSH as a signaling molecule for turning on its virulence [9,20]. In the intracellular state, host or external GSH triggers increased bacterial GSH concentrations. Bacterial endogenous GSH allosterically binds to PrfA, the master virulence transcriptional regulator, and acts as the activating co-factor for PrfA [9,20]. GSH binding to PrfA stabilizes the helix-turn-helix motif of PrfA in an ordered and active conformation, thus priming PrfA for DNA binding [21,22]. This is in fact a common feature of the Crp/Fnr family of transcriptional regulators that similarly bind to allosteric effector molecules to enable DNA binding.

However, it remains unclear how GSH-bound PrfA can be regulated. It is known that a combination of environmental and endogenous cues converges on PrfA to affect its activation. One of them is the growth of the bacteria in rich media, which suppresses PrfA activity. However, adding activated charcoal or adsorbent resin into the rich media reverses and strongly activates PrfA [23]. Through a transposon screen, Krypotou et al. found that nutritional peptides in rich media were transported through the bacterial Opp transport system into the bacteria and competitively bound to PrfA, thereby excluding GSH from binding [24]. At the same time, cysteine containing peptides were scavenged by the transport system that could increase GSH concentration. Therefore, the balance of inhibitory and activating oligopeptides regulate PrfA induction levels through the binding availability for GSH [24].

*P. aeruginosa* strains defective in GSH synthesis exhibit attenuated virulence in several models of infection, including *Caenorhabditis elegans* nematode [25] and *Drosophila melanogaster* fruitfly [26]. However, conflicting results have been reported in the murine models. *P. aeruginosa ΔgshA, ΔgshB* and *ΔgshAΔgshB* double mutants defective in GSH synthesis were attenuated in a mouse model of acute pneumonia [27] yet the *ΔgshA* mutant was equally virulent as the wildtype in surgical wound, abscess and acute burn wound murine infection models [28]. In another report, a *ΔgshA* transposon mutant was found to be enriched in a murine wound model [29]. It is important to note that some of these studies reporting conflicting phenotypes did not complement the mutation with a wildtype copy of the gene [28,29]. Therefore, it is possible that the inconsistency is due to non-specific or polar effects instead of the loss of GSH synthesis. However, several studies which complemented their GSH synthesis mutants, had also reported contradictory data [26,27,30]. For instance, twitching motility, another important virulence factor for *P. aeruginosa*, was also identified to be impaired in several reports [26,27] although a *ΔgshA* transposon mutant revealed no defects in twitching motility [30]. These ambiguities could be attributed to the variability of *P. aeruginosa* laboratory strains which exhibit genomic and phenotypic diversity [31]. While phenotypic characterization of the role of GSH synthesis in *P. aeruginosa* virulence is less clear, a more quantitative approach via transcriptomic analyses of the *ΔgshAΔgshB* mutant revealed that GSH regulates the expression of three categories of virulence-related genes [27]. These three categories include type IV pili biogenesis genes known to contribute to twitching motility, T6SS, a known virulence factor with diverse functions [27,[32], [33], [34]] and T3SS. T3SS was downregulated in the double *ΔgshAΔgshB* mutant and its expression could be rescued by addition of exogenous GSH [27]. In wildtype bacteria, addition of GSH also upregulated T3SS. The authors showed that GSH upregulates T3SS gene expression via the global transcription factor Vfr, which acts on the T3SS central regulator ExsA [27]. Mutations in the cysteine residues on Vfr revealed the involvement of all 5 cysteine residues in the sensing of GSH [27]. The redox state of the cysteines in Vfr isolated from wildtype bacteria were shown to be in the reduced state. When Vfr was oxidized with hydrogen peroxide, it could be reduced by GSH *in vitro*, suggesting that GSH synthesis in *P. aeruginosa* is important for maintaining the redox state of Vfr for modulation of T3SS expression [27]. While the regulatory mechanism of Vfr is somewhat similar to VirA, where an inactive regulatory factor is activated via reduction by GSH, it is important to note that *P. aeruginosa* relies on bacterial GSH rather than host intracellular GSH as is the case for *B. pseudomallei*. Furthermore, one would assume that under normal conditions, the bacteria will be producing GSH and Vfr would be in a reduced state. As *P. aeruginosa* T3SS is induced by several conditions including low Ca^2+^ and host cell contact through ExsA [[35], [36], [37]], the redox control of T3SS via Vfr could be another layer of modulation where oxidizing condition could dampen T3SS induction. At this moment, the mechanism of redox control of Vfr is not known. It is possible that pKa of each cysteine in Vfr is different depending on its surrounding context and has a different propensity to be reduced by GSH. It would be interesting to determine what conditions favor all 5 cysteines to be in the reduced state for Vfr to be fully active, versus when some of them may be differentially modified e.g. through S-glutathionylation or S-nitrosylation.

**Fig. 1:**
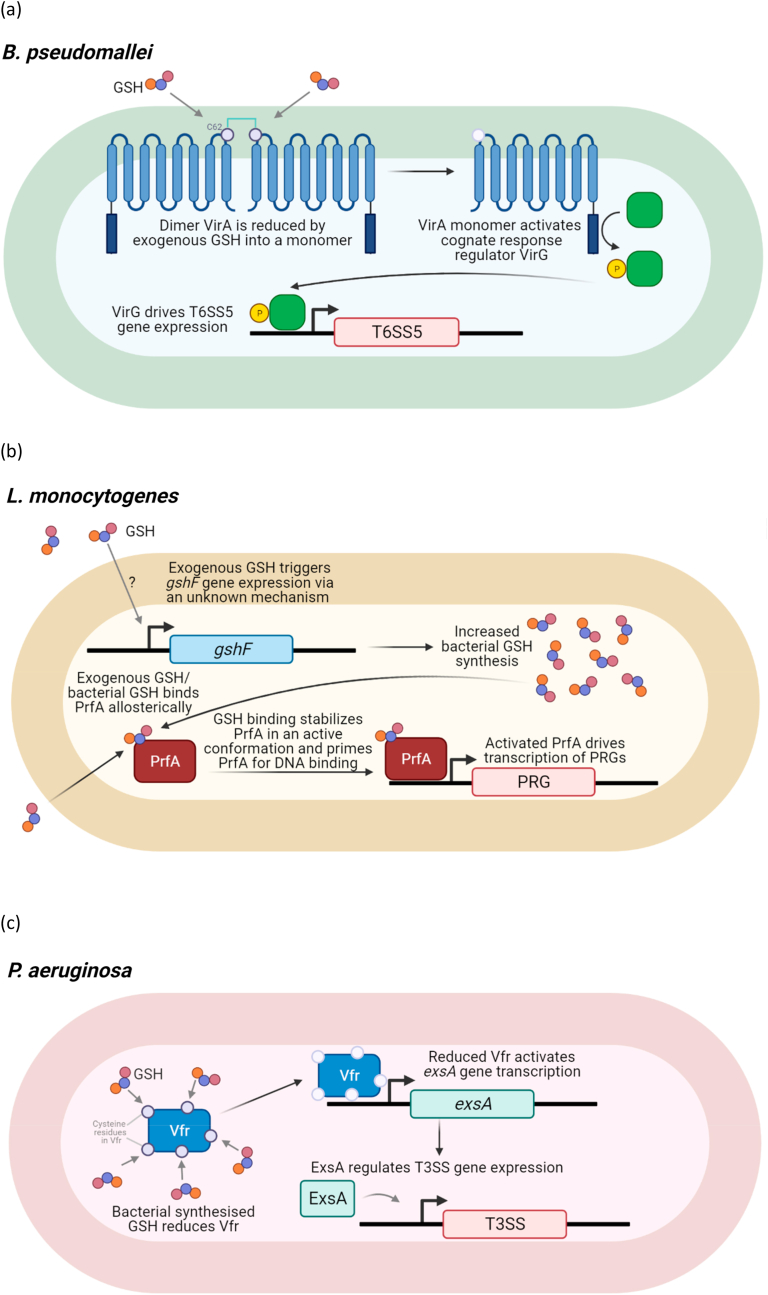
GSH induction of virulence genes during *B. pseudomallei*, *L. monocytogenes* and *P. aeruginosa* infection (a) In *B. pseudomallei*, exogenous GSH reduces VirA, a histidine kinase sensor present on the bacterial inner membrane. Reduction of cysteine residue 62 (C62), predicted to be present at the periplasm, results in a switch from a dimer into a monomeric form. Monomeric VirA activates T6SS5 gene expression via its cognate DNA response regulator VirG. (b) In L. *monocytogenes*, exogenous GSH drives *gshF* expression by an unknown mechanism and increases bacterial GSH synthesis. Exogenous GSH and bacterial synthesized GSH binds allosterically to master virulence regulator PrfA. GSH binding stabilizes PrfA in an active confirmation which primes PrfA for DNA binding. Active PrfA activates the transcription of PrfA-regulated genes (PRGs). (c) In *P. aeruginosa*, bacterial synthesized GSH reduces all 5 cysteine residues (cysteine residues at position 20, 38, 97, 156, and 183) on global transcription factor Vfr. Reduced Vfr activates *exsA* gene transcription. ExsA, in turn, regulates T3SS expression.

In the examples of *B. pseudomallei*, *L. monocytogenes* and *P. aeruginosa,* GSH acts by reducing disulfide bonds or as an allosteric activator of a transcriptional regulator directly controlling virulence genes (Fig. 1). However, GSH has also been shown to directly control virulence function through post-translational modification (PTM).

## 3 Glutathionylation as a control of bacterial virulence

Glutathionylation is a PTM where GSH is added to cysteine residues in a protein. PTMs modulate the structure and function of proteins and thus may be important for the function of virulence related proteins.

Listeriolysin O (LLO), a major virulence factor of *L. monocytogenes*, is a pore-forming hemolytic toxin enabling *L. monocytogenes* to escape from phagolysosomal killing. LLO retrieved from *L. monocytogenes* grown in broth culture were found to be naturally glutathionylated at cysteine residue 484 [38]. In the presence of GSH, hemolytic activity of LLO *in vitro* was eliminated [38]. The modification of LLO at cysteine residue at position 484 to alanine (LLO^C484A^) rendered LLO insensitive to GSH, where LLO^C484A^ retained full hemolytic activity despite the presence of GSH [38]. S-glutathionylation of LLO was shown to prevent association of LLO to its target membrane, thereby inactivating the protein [38]. The compartmentalisation of LLO activity has been previously demonstrated to be important for virulence. Disruptions in the optimal activity of LLO in the phagosome and minimized activity in the host cytoplasm reduced virulence [39]. Thus, S-glutathionylation serves as an additional control to limit LLO's cytolytic activity in the phagosome.

*Yersinia pestis* LcrV, the cap protein of T3SS, is glutathionylated at cysteine residue 273 (C273) [40]. Glutathionylation of LcrV at C273 enhances bacterial virulence as mice and rats infected with *Y. pestis lcrVC273A* mutant, which cannot be glutathionylated, survived better than those infected with wildtype bacteria [40]. The rate of T3SS secretion of effectors is moderated by LcrV glutathionylation [40]. Glutathionylation of LcrV also promotes interaction of LcrV with ribosomal protein S3 (RPS3) which is involved in the regulation of DNA repair, apoptosis and innate immune responses [40]. The association of RPS3 with glutathionylated LcrV suppresses apoptotic cell death and triggers the release of inflammatory cytokines IL-1β and IL-18. IL-1β and IL-18 have been shown to contribute to disease pathology [41].

In *Streptococcus mutans*, S-glutathionylation of cysteine residue 41 (C41) in a thioredoxin-like protein (Tlp) was found to be important for interspecies competition [42]. Relative abundance of *S. mutans tlp-C41A* mutant in the biofilm was reduced to 20.9% as compared to wildtype *S. mutans* (34.7%) in a tri-species biofilm containing *S. mutans*, *Streptococcus sanguinis* and *Streptococcus gordonii* [42]. Interestingly, *Δtlp* only had a mild reduction in relative abundance (32.7%) within the tri-species biofilm. As compared to wildtype, *S. mutans tlp-C41A* and *Δtlp* mutants were both more susceptible to hydrogen peroxide (H_2_O_2_), which was produced by *S. sanguinis* [42]. However, *S. mutans Δtlp* mutant was about 2000-fold less susceptible to H_2_O_2_ compared to *tlp-C41A*. It is possible that the complete loss of Tlp triggers compensatory oxidative resistance mechanisms. Regardless, the decreased abundance of *S. mutans tlp-C41A* mutant in the tri-species biofilm and higher susceptibility of *tlp-C41A* mutant to H_2_O_2_ suggests that S-glutathionylation of Tlp C41 is likely important for Tlp to function as a thioredoxin in *S. mutans*, contributing to resistance against oxidative stress and interspecies competition. Additionally, *S. mutans tlp-C41A* mutant in a rat model of dental caries produced less severe carious lesions on all molar surfaces, indicating that glutathionylation of Tlp has a role in virulence [42].

## 4 Role of GSH in biofilm formation and disruption

Biofilms are organized communities of microorganisms attached to an abiotic or biotic surface often embedded in a matrix. Biofilm formation is associated with enhanced resistance against antibiotics and also virulence [[43], [44], [45]]. The role of bacterial GSH synthesis in biofilm formation has been best documented in *P. aeruginosa*. Phenotypic examinations of biofilm formation via crystal violet staining were contradictory even though the same laboratory strain was utilized, with reports of GSH synthesis mutants increasing [26] and decreasing [27,30] biofilm formation. In particular, the *ΔgshA* transposon mutant in minimal media had decreased growth rate compared to the wildtype, indicating that culture conditions were critical [30]. Interestingly, *P. aeruginosa ΔgshA* mutants had decreased pyocyanin production [26,30]. Pyocyanin has been reported to mediate aggregation of *P. aeruginosa* and promote the release of extracellular DNA, a biofilm component. As discussed in the previous section, GSH upregulates type IV pili and T6SS gene expression [27], both of which have been reported to affect biofilm formation [[32], [33], [34]]. Since the use of GSH synthesis mutants could result in secondary effects which affect biofilm formation, the issue could be complex. Additionally, biofilm architecture instead of crude biofilm biomass as measured by crystal violet assay should be examined. In a separate study, the *ΔgshAΔgshB* mutant in a single-species biofilm had comparable growth and biofilm biomass to the wildtype *S. mutans* strain [46]. However, the *ΔgshAΔgshB* mutant was differentially organized within the biofilm with formation of microcolonies, compared to the wildtype strain which were evenly distributed [46]. The *ΔgshAΔgshB* mutant had enhanced extracellular polysaccharide (EPS) production and upregulated expression of EPS synthesis genes such as glucosyltransferases *gtfB*, *gtfC*, and *gtfD* [46]. The authors speculate that the increased EPS production and microcolony formation served as protection from oxidative stress. Thus, analysis of the crude biofilm biomass is not sufficient for one to make conclusions on the changes in biofilm formation. Given the complex composition and structure of biofilms, caution should be exercised in the interpretation of bacterial synthesized GSH and their effects on biofilm formation.

On the other hand, exogenous GSH has been described in many studies to be capable of disrupting biofilms of various bacterial strains and improving antibiotic efficacy. The effects of GSH disruption of biofilms have been reported at high concentrations, ranging from 1 mM to 30 mM and for monomicrobial biofilms of *P. aeruginosa*, *S. pyogenes*, *S. aureus*, *K. pneumoniae*, *Enterobacter* sp., *E. coli* and *A. baumannii*, including clinical and multidrug resistant (MDR) strains [[47], [48], [49]]. Several studies also investigated the potential mode of action of exogenous GSH on biofilm disruption and the enhancement of antibiotic effectiveness, examining transcriptome changes [48] or the effect on MDR efflux pumps or beta-lactamase activity post-GSH treatment [49]. These findings, however, should be examined prudently as GSH at high concentrations is highly acidic (i.e. 20 mM of GSH has a pH of 3.92 and 3.89 when dissolved in Luria Broth (LB) or Phosphate buffered saline (PBS) respectively) [3,47]. A study reported that 30 mM GSH at neutral pH of 7.2 did not decrease the viability of MDR *A. baumannii* biofilm (76–94%) whereas 30 mM GSH at unbuffered intrinsic pH significantly decreased biofilm viability (16–38%) [47]. The intrinsic acidic pH of GSH was also shown to destabilise and cleave dsDNA, which likely contributes to biofilm disruption [47]. We highlight the importance of considering acidity when performing experiments with exogenous GSH. It remains to be clarified if GSH does indeed play a role in biofilm disruption apart from its acidity.

## 5 GSH catabolism to hydrogen sulfide (H_2_S) and its role in virulence

Although H_2_S has been described to protect bacteria from oxidative stress, it is also involved in the virulence of several bacteria [[50], [51], [52], [53]]. The production of H_2_S via GSH catabolism for virulence has been reported for *Treponema denticola*, an oral spirochete bacterium associated with periodontal disease. *T. denticola* utilizes a three-step pathway involving γ-glutamyltransferase (*ggt*) for catabolism of GSH to H_2_S [54]. The high levels of H_2_S in periondontal pockets, hemoxidation and hemolysis activity against red blood cells and ability to induce apoptotic cell death are thought to contribute to periodontal pathology [[55], [56], [57]]. Exogenous addition of GSH led to H_2_S production as well as hemolysis and hemoxidation activities [58]. In a separate study, *Δggt*, which does not produce H_2_S, induced lower gingival fibroblast cell death compared to wildtype bacteria [51]. Addition of exogenous GSH led to higher levels of H_2_S production, which corresponded with higher levels of gingival fibroblast death [51]. Although the acidity of exogenous GSH was not considered in these experiments, the GSH concentration used in the *in vitro* cell death assay was 2 mM, which approximates a pH of >6 in non-buffered solutions. Given that the physiological pH of the oral cavity ranges pH 6.2 to 7.6 [59], it is unlikely that the gingival fibroblasts were undergoing cell death due to the slightly acidic pH. Thus, it is conceivable that the higher level of gingival fibroblast cell death was due to GSH conversion to H_2_S. In a murine abscess model, animals inoculated with wildtype *T. denticola* supplemented with GSH experienced larger lesion sizes as compared to animals challenged with *Δggt* and GSH [51]. This shows that GSH conversion to H_2_S contributes to soft tissue destruction and plays a role in *T. denticola* virulence. It remains to be investigated whether other pathogenic bacteria utilize GSH for virulence through its conversion to H_2_S. However, since H_2_S can be derived from other substrates besides GSH, the role of GSH catabolism to H_2_S for virulence is unlikely to be conserved across pathogenic bacteria.

For commensals such as *E coli*, the *tnaA* gene that is annotated as a tryptophanase and reported to break down tryptophan to indole and pyruvate [[60], [61], [62]], has also been reported to be important for catalyzing the metabolism of cysteine to H_2_S [63]. This gene has homologs in many Enterobacteriaceae and one intriguing possibility is that these commensal intestinal bacteria would be able to produce H_2_S due to high concentrations of GSH in the largely anaerobic gut. GSH metabolism has been shown to be important at the intestinal interface [64]. It has been reported that during intestinal dysbiosis, blooms of Proteobacteria occur [65,66]. Besides the H_2_S already produced by colonic gut microbiome, the increase in Proteobacterial species with ability to catabolize GSH to H_2_S could increase total H_2_S concentrations in the gut. H_2_S can be converted to thiosulfate in the gut to prevent toxic build-up that is injurious to cells normally. But during mild inflammation, thiosulfate could be oxidized to tetrathionate, which enteric pathogens such as *Salmonella* could use as a terminal electron acceptor for respiration [67]. Therefore, it will be interesting to explore whether GSH catabolism to H_2_S by opportunistic bacterial species in the gut could impact gut health.

## 6 GSH and its functional equivalents’ contribution to virulence

MSH and BSH are the GSH functional equivalent in some bacteria which do not produce GSH. MSH is the major LMW thiol produced by actinobacteria which includes Mycobacteria [68]. On the other hand, BSH, the α-anomeric glycoside of L-cysteinyl-d-glucosamine with l-malic acid, is an abundant thiol produced by some firmicutes including clinically relevant pathogens *Bacillus anthracis*, *Bacillus cereus*, *Staphylococcus aureus*, *Staphylococcus saprophyticus*, and *S. agalactiae* [69]. Notably, *S. agalactiae* synthesizes GSH that was shown to be important for its pathogenesis [70]. Mice infected with the *ΔgshAB* mutant defective in GSH synthesis in a sepsis model of infection had higher survival and reduced bacterial loads in the blood [70]. *S. agalactiae ΔgshAB* mutants exhibited a minimal to moderate protection against ROS stressors H_2_O_2_ and HOCl indicating that GSH synthesis in *S. agalactiae* neutralizes ROS stress [70]. It is possible that *S. agalactiae* GSH synthesis contributes to its pathogenesis by enhancing resistance against oxidative stress. It is not known whether GSH could be involved more directly in *S. agalactiae* virulence and the role of GSH in *S. agalactiae* pathogenesis remains to be clarified.

*Mycobacterium tuberculosis* utilizes the inhibition of phagosome maturation and resistance to acidic pH as virulence strategies to establish chronic infections [71]. Acidic pH was shown to affect cytoplasmic MSH redox potential which is sensed by the WhiB3 redox sensor for modulating virulence gene expression [72]. This includes the upregulation of polyketide biosynthesis genes and ESX-1 secretion, which restricts phagosomal acidification and induces phagosomal rupture respectively [72]. This could explain why the *M. tuberculosis ΔmshA* mutant defective in mycothiol synthesis has lower expression of ESX-1 secretion system genes [73], and significantly lower intracellular bacterial loads in murine macrophages at later timepoints of a low dose of infection [74]. This demonstrates that *M. tuberculosis* utilizes the MSH redox system to respond to the acidic pH of the phagosomal compartment for its virulence. Aside from ESX-1 secretion system genes, a lack of MSH in *M. tuberculosis* results in differential expression of VapC toxins, metabolic genes and cytochrome biogenesis [73]. It remains to be investigated how MSH deficiency contributes to these transcriptome changes and whether the differential gene expression contributes to virulence and fitness for *M. tuberculosis* infection.

As discussed in an earlier section, biofilm formation is a virulence factor for pathogenesis of various bacteria. *Mycobacterium smegmatis* form pellicular biofilms at the air-media interface [[75], [76], [77]]. *M. smegmatis* mutants defective in MSH synthesis have decreased biofilm formation compared to the wildtype bacteria [78]. This suggests that MSH synthesis is required for effective formation of biofilms by *M. smegmatis* although it remains unclear how MSH influences the process.

BSH has been demonstrated to play a role in survival of *S. aureus* in host cells. *S. aureus ΔbshA* mutant defective in BSH synthesis survived less well than the wildtype strain in whole blood containing neutrophils, macrophages, and complement [79]. It is important to note that *S. aureus* is a Gram-positive bacterium which is believed to be resistant to complement killing via membrane attack complex due to the thick peptidoglycan cell wall [80]. Thus, the reduced bacterial numbers of *ΔbshA* retrieved from whole blood [79] is likely due to poorer survival in the midst of blood cells rather than increased susceptibility to complement. More direct evidence was shown in another study which examined members of the *S. aureus* NCTC8325 lineage that is incapable of producing BSH [81]. Reconstitution of BSH synthesis in *S. aureus* strain 8325-4 resulted in higher bacterial loads in murine macrophages and human upper airway epithelial cells [81]. A study characterising YpdA, a putative BSH disulfide reductase that recycles oxidized bacillithiol disulfide (BSSB) to reduced BSH, also provided evidence that BSH enhanced survival of *S. aureus* in host cells [82]. YdpA mutant, which had lower levels of BSH and higher levels of BSSB, had reduced survival in human neutrophils [82]. Conversely, cells overexpressing YdpA survived better than cells with the empty vector control [82]. Diphenyleneiodonium (DPI) treatment abrogated the poorer survival of YpdA mutant in human neutrophils [82]. DPI was used as an inhibitor of oxidative burst, leading the authors to conclude that BSH functions to protect *S. aureus* from oxidative burst in human neutrophils [82]. However, aside from inhibiting nitric oxide synthesis, DPI has been demonstrated to possess antibacterial activity [83] and induce oxidative stress in murine glial cells [84]. Thus, stronger evidence is required to confirm if BSH indeed confers protection to *S. aureus* via resistance to oxidative burst for enhanced survival in the human neutrophils. On a related note, transcriptome analyses revealed that *S. aureus ΔbshA* mutants had differential gene expression of a variety of genes involved in metabolism, transporters, transcription regulators and virulence [79]. So far, the documented role of MSH and BSH in virulence is mainly indirect and could be due to protection against oxidative stress encountered in biofilm or mediated by the host. It would be exciting to see if they are involved in redox regulation of virulence regulators as seen in *B. pseudomallei*, *L. monocytogenes* and *P. aeruginosa*.

## 7 GSH as an immune modulator in bacterial infection

Much of the work done in examining the effect of GSH on the immune response against bacterial infections is in tuberculosis (TB) and melioidosis.

GSH has been previously shown to have direct antimycobacterial effects [[85], [86], [87]] likely due to reductive stress experienced by the microbes. Mycobacteria lack GSH and possess the alternative thiol, mycothiol, to regulate redox homeostasis. Therefore, physiological concentrations of GSH (in millimolar) inside the macrophages can cause reductive stress leading to growth inhibition of *M. tuberculosis* [[85], [86], [87]]. Both GSH and N-acetylcysteine (NAC) were also reported to diminish TB pathology and inflammation [[88], [89], [90], [91], [92]]. GSH's and NAC's potent anti-inflammatory effects are thought to be through dampening the activation of nuclear factor-kB (NF-kB) as well as the specific inhibition of other proinflammatory cytokine synthesis [[93], [94], [95]]. In both experimental animal models as well as clinical studies, NAC has been shown to have a protective effect against liver damage from anti-TB medications [96]. Furthermore, Vilchèze et al. demonstrated that the synergistic combination of cysteine or other small thiols with first-line TB antibiotics such as isoniazid or rifampicin prevented the formation of drug-tolerant and drug-resistant *M. tuberculosis* cultures by shifting the menaquinol/menaquinone balance toward a reduced state [97]. This stimulates bacterial respiration and converts persister cells to metabolically active cells which become susceptible to antibiotics [97]. Teskey et al. further showed that NAC with suboptimal levels of isoniazid and rifampicin could also clear *M. tuberculosis* infection from *in vitro* derived granulomas [98].

**Fig. 2:**
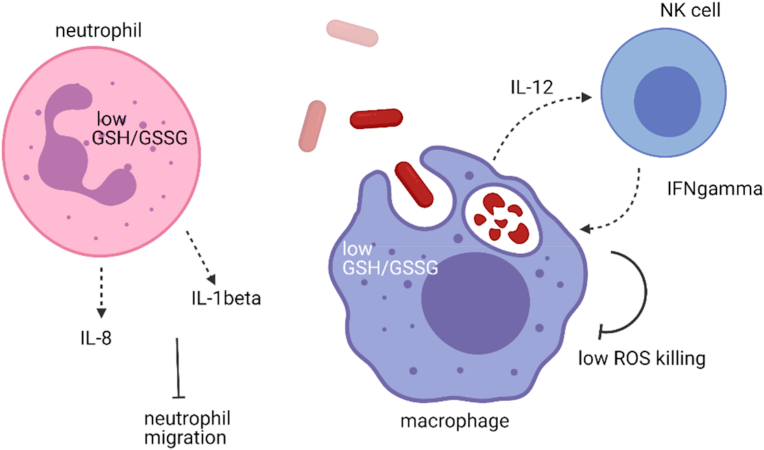
GSH modulation of the immune response against *B. pseudomallei* infection. A low GSH:GSSG ratio as depicted leads to low IL-1β and IL-8 production in the neutrophils, as depicted by dotted arrows. The low production of these cytokine and chemokine in response to infection impairs neutrophil migration. The low GSH:GSSG ratio in monocytes and macrophages results in impaired IL-12 production (dotted arrows) and leads to a corresponding decrease in IFNγ production in NK cells (dotted arrows). IFNγ is necessary for activating macrophages to be microbicidal and efficient in killing of intracellular bacteria. Created with BioRender.com.

Melioidosis, a disease caused by *B. pseudomallei*, shows some similarity to TB in that both are caused by intracellular bacteria and infections could turn chronic. Even more so than TB, the most important risk factor in melioidosis is Type 2 diabetes, where up to 60% of melioidosis patients are diabetic [[99], [100], [101], [102]]. In trying to discover the underlying susceptibility to disease in the diabetics, it was previously reported that PBMCs obtained from diabetic patients with poor glycemic control (HbA1c > 8) had a GSH deficiency, where ratios of GSH to oxidized glutathione (GSSG) were decreased as compared to healthy individuals and diabetics with good glycemic control although total concentrations did not appear to differ [103]. Lowered GSH:GSSG ratios correlated with impaired IL-12 and IFN-γ production and poor intracellular bacterial control in macrophages [103]. IL-12p70 consists of two protein subunits, IL-12p35 and IL-12p40. Low IL-12 production is contributed by a reduction in the transcription of the IL-12p35 subunit, which is the rate limiting step in IL-12 production [103]. There is no direct evidence indicating how GSH affects IL-12p35 transcription. IL-12 then induces IFN-γ production from natural killer (NK) cells , and IFN-γ in turn activated monocytes and macrophages to increase bactericidal activity. GSH deficient mice treated with DEM or buthionine sulfoximine (BSO) succumbed to *B. pseudomallei* infection at rates faster than those without treatment [103]. The reason that could explain the poor infection outcome of GSH deficient mice is that the GSH:GSSG ratio directly affected the production of IL-12 from macrophages and this caused a reduction of IFN-γ production from NK or T cells, which could in turn activate the microbicidal activity of macrophages to kill the intracellular bacteria [104]. In a separate study, the ratio of GSH:GSSG in polymorphonuclear neutrophils (PMNs) isolated from diabetic individuals receiving glibenclamide therapy (a treatment for diabetics in the region the study was conducted) was lower than healthy or diabetic individuals who did not receive glibenclamide. The reduction in GSH in the neutrophils correlated with decreased IL-8 and IL1-β production in response to *B. pseudomallei* infection [105]. Supplementation with GSH or GSH precursor NAC prior to *B. pseudomallei* infection in the glibenclamide-treated neutrophils restored cytokine responses and improved neutrophil migration [106]. These studies support the role of GSH in modulating protective immune responses against *B. pseudomallei* (Fig. 2). One caveat with all these studies using addition of NAC, GSH or DEM to modulate intracellular GSH is that the GSH:GSSG couple may not be the only redox pairs affected within the cells. Unless one is able to identify how GSH specifically modifies genes or effectors, the effect of GSH could be through indirect effects on immune response genes.

While it may seem intuitive to boost intracellular GSH levels to reduce susceptibility towards *B. pseudomallei* infection, targeting GSH:GSSG ratio is not straightforward. A previous attempt to enhance the IL-12 protective response against *B. pseudomallei* via oral supplementation of NAC in diabetic patients resulted in only an increase in free GSH and GSSG but not the overall GSH:GSSG ratio [104]. *B. pseudomallei* infection of the isolated PBMCs also revealed unaltered IL-12, IFN-γ responses and intracellular bacterial loads [104]. This could be because the underlying cause of high oxidative stress in diabetes is not removed, and more exogenous GSH is simply converted to GSSG without a change in the redox ratio. Given that host GSH turns on *B. pseudomallei* virulence [10], there is the additional complexity where modulating GSH levels could potentially boost the virulence of the pathogen. However, since diabetics with lower GSH:GSSG levels are more susceptible to *B. pseudomallei* infection, it is likely that a low GSH threshold exists for triggering the T6SS5 virulence program (cytoplasmic concentrations are in millimolar range even in diabetics) and that boosting GSH levels benefit the host more than the bacterium.

In Lyme disease caused by the spirochete *Borrelia burgdorferi*, GSH metabolism was increased 10-fold in macrophages upon infection [107]. The increased GSH levels resulted in increased IL-1β processing and secretion and decreased TNFα mRNA translation. The authors deduced these alterations could be through glutathionylation. However, the targets of these modifications are unknown. Nevertheless, this study shows that bacterial infection can modify GSH metabolism in the host that translates into modulation of cytokine responses against the infection.

In fact, ROS responses triggered by infection alter GSH metabolism in T cells. GSH was shown to be essential for T cell effector function through metabolic programming [108]. GSH deficient T cells initially underwent normal activation but could not meet their increased energy and biosynthetic requirements [108]. GSH deficiency compromised the activation of mammalian target of rapamycin-1 (mTOR) and expression of NFAT and Myc transcription factors, abrogating the energy utilization and Myc dependent metabolic reprogramming that allows activated T cells to switch to glycolysis and glutaminolysis [108]. As a result, this impacts anti-viral immunity [108]. The dependence on GSH to mediate T cell effector functions will similarly affect adaptive immune response to bacterial infections. A very recent report also demonstrated how metabolism affects T regulatory cell maintenance. Presence of GSH limits serine metabolism and maintains FoxP3 expression, necessary for T regulatory cell function [109], which would be important for preventing too much inflammation. Although not directly related to bacterial infections, this is another piece of evidence on how GSH has broad effects on the immune system that can impact immune function when under attack by pathogens. Furthermore, activity of macrophages are also regulated by GSH, as reviewed [110]. Besides the obvious role of GSH as an antioxidant in these cells capable of much ROS production, GSH through glutathionylation on the enzyme peroxiredoxin-2 could drive TNFα release, and inhibit caspase-1 activation also through glutathionylation [110].

## 8 Conclusion

It is perhaps not surprising that GSH, being the most important redox systems in many bacteria to maintain metabolism and homeostasis, play a role in bacterial pathogenicity by maintaining optimal bacterial growth and survival. What has been less obvious is how a handful of bacteria use GSH as a reducing moiety or allosteric regulator of their transcription factors to directly upregulate virulence pathways. Some bacteria could also control many post-translational responses via glutathionylation of bacterial virulence factors. Furthermore, GSH modulates the immune system in many complex ways that influence infection outcomes. GSH can modify redox sensitive transcription factors in both bacteria and host cells to effect transcriptional changes. Many of these changes could be via glutathionylation of enzymes and transcription factors, and through changes in energy metabolism that are only now beginning to be explored. These complex changes will influence how an infection progresses in the mammalian host. The research area on how GSH modulates immune responses to infections is currently understudied. We predict that with further investigations, more examples will be uncovered to demonstrate how diverse bacterial pathogens could use GSH to regulate their virulence, and how the host could use GSH to modulate its response against these bacterial incursions.

## Declaration of competing interest

None.
